# A Sequence Obfuscation Method for Protecting Personal Genomic Privacy

**DOI:** 10.3389/fgene.2022.876686

**Published:** 2022-04-13

**Authors:** Shibiao Wan, Jieqiong Wang

**Affiliations:** ^1^ Center for Applied Bioinformatics, St. Jude Children’s Research Hospital, Memphis, TN, United States; ^2^ Department of Radiology, University of Pennsylvania, Philadelphia, PA, United States

**Keywords:** genomic privacy, obfuscation methods, DNA generalization lattice, MegaBLAST, sequence similarity, clustering, machine learning, IterMegaBLAST

## Abstract

With the technological advances in recent decades, determining whole genome sequencing of a person has become feasible and affordable. As a result, large-scale individual genomic sequences are produced and collected for genetic medical diagnoses and cancer drug discovery, which, however, simultaneously poses serious challenges to the protection of personal genomic privacy. It is highly urgent to develop methods which make the personal genomic data both utilizable and confidential. Existing genomic privacy-protection methods are either time-consuming for encryption or with low accuracy of data recovery. To tackle these problems, this paper proposes a sequence similarity-based obfuscation method, namely IterMegaBLAST, for fast and reliable protection of personal genomic privacy. Specifically, given a randomly selected sequence from a dataset of genomic sequences, we first use MegaBLAST to find its most similar sequence from the dataset. These two aligned sequences form a cluster, for which an obfuscated sequence was generated *via* a DNA generalization lattice scheme. These procedures are iteratively performed until all of the sequences in the dataset are clustered and their obfuscated sequences are generated. Experimental results on benchmark datasets demonstrate that under the same degree of anonymity, IterMegaBLAST significantly outperforms existing state-of-the-art approaches in terms of both utility accuracy and time complexity.

## 1 Introduction

**FIGURE 1:**
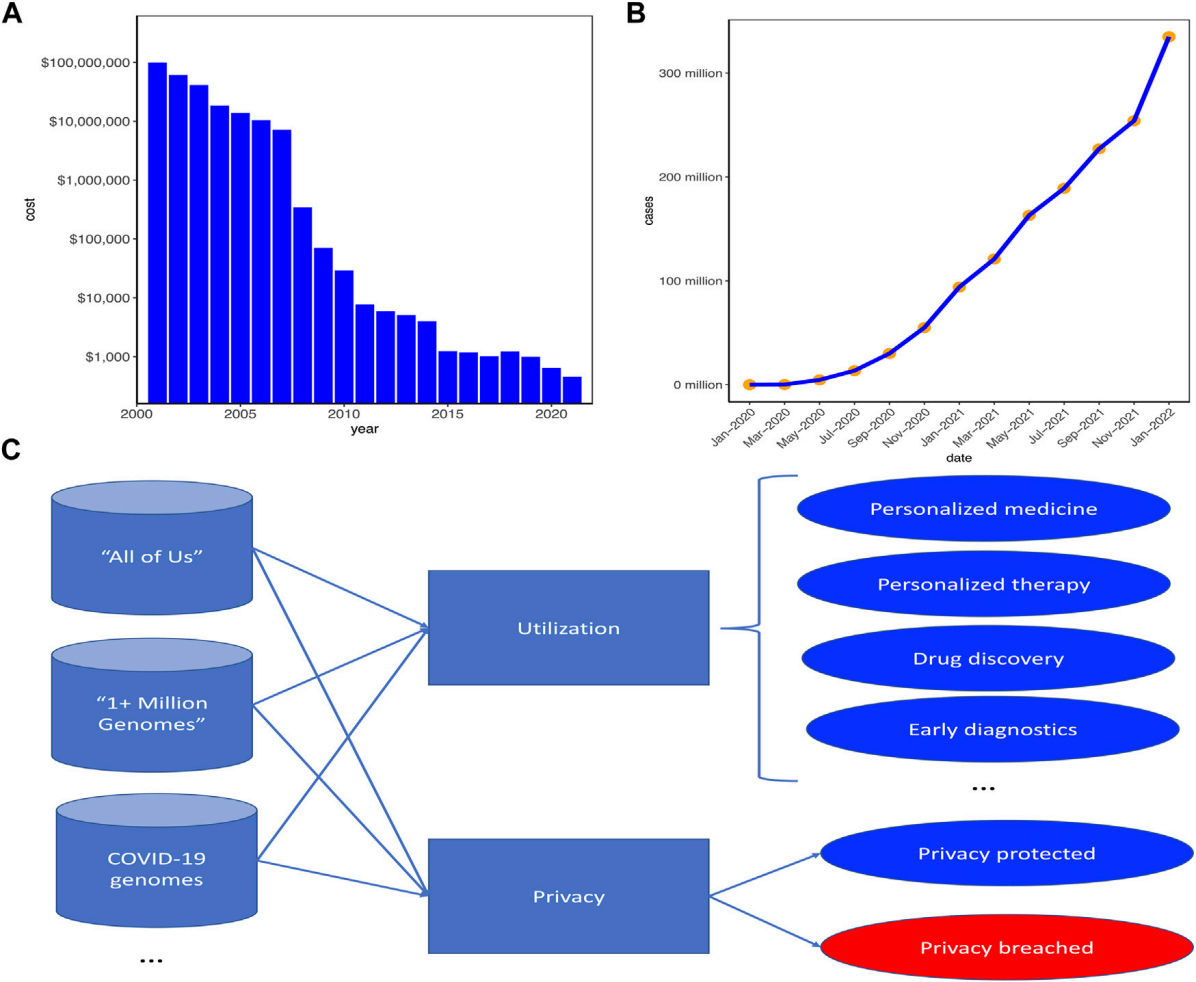
Current status of personal genomic data for utilization and privacy. **(A)** The whole genome sequencing (WGS) cost decreased significantly with the technological advances in recent decades. **(B)** The number of COVID-19 cases increased significantly in these 2 years and concurrently the number of personal genomic data would increase. **(C)** Large-scale projects have been launched for betterment of human healthcare while simultaneously posing serious challenges on protecting individual genomic privacy. “All of Us” (https://allofus.nih.gov/) was launched by US and “1 + Million Genomes” (https://digital-strategy.ec.europa.eu/en/policies/1-million-genomes) was initialized by the European Union. Blue circles represent good benefits of genomic data utilization or privacy well proteted, whereas the red circle represents the challenge of genomic data privacy breached.

With the technological advances in recent decades, the cost of sequencing a whole human genome has been dramatically decreased^1^. As can be seen from Figure 1A, when the first human genome was sequenced in 2001, the total cost was around 300 million USD. However, in 2006, the cost was decreased to 14 million USD and in 2016, the cost was below 1500 USD. With the feasibility and affordability of whole genome sequencing (WGS) for personal tests, large swathes of personal genomic data have been generated.

As a result, recent decades have witnessed the widespread applications of genomic high-throughput technologies in personalized healthcare (Chute and Kohane, 2013), with which large-scale personal genomic data are produced and collected for genetic medical diagnoses and new drug discovery. Moreover, individuals become more willing to share their genomic data on some health-related websites [e.g., OpenSNP (https://opensnp.org/) (Greshake et al., 2014)] to learn their predispositions to genetic diseases and their ancestries (Humbert et al., 2013). Besides, with the COVID-19 pandemic entering the third year of upending life around the world and more than 300 millions of people have been infected (Figure 1B), hundreds of thousands if not millions of COVID-19 patients have their genomes sequenced to help scientists and researchers to unravel the genetic mechanisms of the SARS-CoV-2, the virus causing the COVID-19 pandemic. Moreover, the US and Europe have launched their respective plans (i.e., “All of Us” (https://allofus.nih.gov/) for US and “1 + Million Genomes Initiative” (https://digital-strategy.ec.europa.eu/en/policies/1-million-genomes) to sequence at least one million human genomes to unlock genetic mysteries (Figure 1C).

All of these events significantly boost the rapid accumulations of personal genomic data in huge size. On the positive side, the large-scale individual genomic data demonstrate the advancement of biomedical technologies and will bring tremendous benefits to biomedicine and patient healthcare as well as accelerate the progress of personalized medicine, personalized therapy, drug discovery, early diagnostics and prevention, etc. On the negative side, however, these events simultaneously pose serious challenges to the protection of personal genomic privacy. Actually, the genomic information of an individual can be as personally indicative as his/her fingerprint, if not more revealing (Leonard et al., 1972). The genomic information is highly at risk of being abused to affect employment, insurance status, etc (Clayton, 2003). Due to the large size and rich information of personal genomic data, it is much more difficult to protect the genomic privacy of an individual than other sensitive information (such as social security numbers and names) that can be securely protected by encryption (Malin and Sweeney, 2004). Therefore, it is highly required to develop efficient and fast methods for protecting genomic privacy while utilizing the genomic information for specifically designated purposes, such as medical diagnosis and new drug discovery.

Existing approaches for genomic privacy protection can be roughly divided into three categories: 1) cryptology-based methods (Kantarcioglu et al., 2008; Goodrich, 2009); 2) data de-identification methods (Malin and Sweeney, 2000; Malin and Sweeney, 2004) and 3) data augmentation methods (Lin et al., 2002; Malin, 2005b).

• **Cryptology-based methods** do not disclose raw genomic data while supporting the genomic data mining. However, this kind of methods are not suitable for long-term genomic privacy protection because the cryptographic algorithms can be broken in a comparably shorter time than the personal genomic privacy protection requires (Humbert et al., 2013). Besides, they offer no protection against re-identification (Loukides et al., 2010).
• **Data de-identification methods** tend to remove or encrypt those genomic data-associated identifiers which are also personally specific and sensitive, such as social security numbers or names. Nevertheless, these methods cannot guarantee sufficient privacy protection and are not able to deal with the re-identification problems (Malin, 2005a).
• **Data augmentation methods** achieve the goal of privacy protection by generalizing or obfuscating DNA sequences, which can make each record indistinguishable from each other. With this kind of methods, the privacy of genomic data can be well protected at the expense of limited loss of data utility.

Among the aforementioned methods, a DNA sequence obfuscation method called DNA lattice anonymization (DNALA) (Malin, 2005b) is one of the state-of-the-art approaches. DNALA is based on the famous *k*-anonymity principle (Sweeney, 2002) which uses a generalized sequence to represent *k* aligned DNA sequences after sequence alignment and clustering. In this way, individual sequences within a cluster will not be distinguished. This method can efficiently protect the personal genomic privacy; however, it uses a low-accuracy clustering algorithm called CLUSTALW (Thompson et al., 1994) and a time-consuming sequence alignment technique. Later, Li et al. (2007) proposed a stochastic hill-climbing method to improve the clustering algorithm for better performance. Recently, Li et al. (2012) further reduced the information loss for genomic privacy protection by proposing a maximum-weight matching (MWM) based algorithm. However, these methods are still inefficient and with low accuracy.

To address these problems, this paper proposes a sequence-similarity based obfuscation method, namely IterMegaBLAST, for protecting personal genomic privacy. Unlike previous methods (Malin, 2005b; Li et al., 2007; Li et al., 2012), which use CLUSTALW as the clustering algorithm, IterMegaBLAST uses MegaBLAST (Zhang et al., 2000) for both sequence alignment and clustering. MegaBLAST is a sequence alignment search algorithm which finds highly-similar sequences to the query one. Specifically, given a dataset, we iteratively use MegaBLAST to find homologs within the dataset for randomly selected query sequences. Then, the query sequences and the corresponding homologs are subsequently formed as clusters for further sequence obfuscation. Our results also demonstrate that IterMegaBLAST is much faster and more accurate than the existing state-of-the-art methods under the same degree of privacy protection. IterMetaBLAST is publicly available at https://github.com/shibiaowan/IterMegaBLAST.

## 2 Methods

### 2.1 Problem Statement

Given a dataset of DNA sequences, our objective is to protect the individual-specific genomic information from identification and/or re-identification^2^ as much as possible while the loss of information affecting the data utility is as little as possible. In other words, the genomic privacy is enhanced at the expense of data precision reduction. One of the effective ways is to obfuscate the differential information within a cluster of DNA sequences with high sequence similarity. In this way, the individual-specific privacy information can be preserved while the loss of information is the minimum.

Generally speaking, given a dataset of genomic data ${\left\{Q\right\}}_{i=\left\{1,\ldots ,N\right\}}$ , for which the *i*-th element $Q_{i}$ represents the individual genomic information (e.g., DNA sequence) for the *i*-th person whose sensitive attributes might be identified *via* one or more individual-specific loci by combining with publicly available (yet perhaps anonymized) information (e.g., demographic). *N* is the number of genomic sequences within the dataset of interest. Our purpose is to find an encryption method *f* so that after encryption, i.e., $G_{i}=f\left(Q_{i}\right),i=\left\{1,\ldots ,N\right\}$ , the personal genomic privacy P is not compromised whereas the utility U of the genomic data is conserved as much as possible.

$$\begin{array}{ll} & \underset{f}{\mathrm{arg min}}\overset{N}{\underset{i=1}{\sum }}\frac{U\left(Q_{i}\right)-U\left(G_{i}\right)}{1-P\left(G_{i}\right)}\\ = & \underset{f}{\mathrm{arg min}}\overset{N}{\underset{i=1}{\sum }}\frac{U\left(Q_{i}\right)-U\left(f\left(Q_{i}\right)\right)}{1-P\left(f\left(Q_{i}\right)\right)}, \end{array}$$ (1)

where P(x) and U(x) is the privacy and utility functions for the *x*-th genomic sequence, respectively.

We assume that the utility value after encryption will not surpass that before encryption (i.e., $U\left(Q_{i}\right)\geq U\left(G_{i}\right)$ ), because any encryption method would incur information loss. For simplicity, we consider the output of the privacy function represents the degree of privacy being compromised (suppose the privacy can be quantified). In most cases, we don’t want our (genomic) privacy being compromised as much as possible. In other words, the output of the privacy function should be only binary, i.e., 0 (the privacy is not compromised) or 1 (the privacy is compromised). When the privacy is compromised even after data encryption, i.e., $P\left(f\left(Q_{i}\right)\right)=1$ , Eq. 1 will equal to + *∞*, which is not we want. In other words, we should first find the encryption function *f* that can protect our privacy and based on this condition, we try to minimize the utility loss as much as possible. In this paper, we use an encryption method based on *k*-anonymity (Section 2.3), which is an efficient way to protect the data privacy. In this case, Eq. 1 has been converted into a problem to find a method to maximize the utility value of the encrypted genomic data. In the following sections, we will elaborate our method to simultaneously protect the genomic privacy and maximize the utility value.

Due to their special properties, DNA sequences can not be clustered if without sequence alignment. Therefore, the procedures for an obfuscation method for genomic privacy protection generally include two steps: 1) sequence alignment and clustering; and 2) obfuscation (or anonymization).

### 2.2 MegaBLAST for Sequence Alignment and Clustering

MegaBLAST is a DNA sequence alignment search tool which uses a greedy algorithm (Zhang et al., 2000) to find those highly-similar sequences to the query one. MegaBLAST is optimized to find near identities and can provide functions of both sequence alignment and clustering. Compared to the traditional BLAST algorithm (Altschul et al., 1997), MegaBLAST runs 10 times faster and is particularly efficient to handle much longer DNA sequences.

Therefore, MegaBLAST is very suitable for our case due to the following reasons: 1) the genomic data (i.e., DNA sequences) concerned should be aligned and clustered before obfuscation methods are used; 2) in practical situations, a fast sequence alignment and clustering tool is highly required to deal with a tremendous number of DNA sequences; 3) usually genomic privacy protection should be imposed on datasets of DNA sequences within the same species, which are often with high sequence similarity and MegaBLAST specifically excels in handling highly-similar sequence alignment.

Because MegaBLAST can find a list of homologs^3^ to the query sequence, we can select a certain number (i.e., the *k* defined in Section 2.3) of the top homologs together with the query sequence to form a cluster. Later, obfuscation methods are imposed on each cluster for genomic privacy protection.

### 2.3 *k*-Anonymity

The *k*-anonymity (Sweeney, 2002) was initially proposed to tackle a problem of how to make the individual data-owners indistinguishable while their data are publicly released and remain practically useful. The value *k* refers to the number of individuals (or samples) within a cluster. In other words, the data are originally entity-specific and well-organized which are represented by some semantic categories (or attributes) consisting of a set of values. To prevent the data owners from being re-identified, a typical *k*-anonymity based method uses *generalization*. Generalization methods are based on a linear and unambiguous generalization hierarchy (Malin, 2005b) where the value at the higher level (ancestor) is less-specific than that at the lower-level (child). They replace the value of each individual by a higher-level value *via* the generalization hierarchy rule. For example, we can use “California” to replace “Los Angeles” and “San Diego,” and use “United States” to replace “California” and “New York”. In this way, a released data set processed by a *k*-anonymity method can guarantee that an individual’s record within this data set cannot be distinguished from at least (*k* − 1) other individuals. In other words, the probability of re-identifying an individual based on the data set is no more than 1/*k*. Obviously, a larger *k* will provide better privacy protection. Besides generalization, suppression (Kisilevich et al., 2010) is another way to realize the *k*-anonymity.

### 2.4 Sequence Obfuscation

**FIGURE 2:**
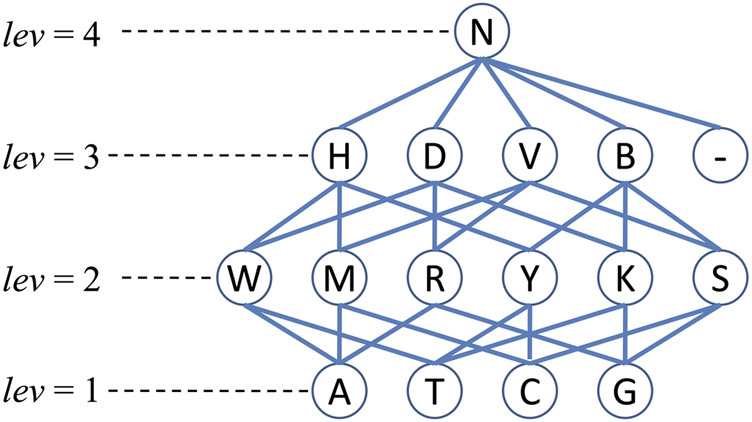
The generalization hierarchy (Malin, 2005b) for sequence or nucleotide obfuscation. Note that *lev* is the level of corresponding nucleotides and the symbol “-” represents the gap.

In this paper, for sequence obfuscation, we used a method proposed in (Malin, 2005b). This method used a generalization hierarchy based on the IUPAC nucleotide representation code (IUPAC-IUB Comm. on Biochem. Nomenclature, 1970). Generally speaking, the basic four nucleotides (A, T, C and G) act as the elements in the 1-st level of the generalization hierarchy; in the 2-nd level, six letters (R, W, M, K , S and Y) are used to represent the six different combinations of any two nucleotides in the 1-st level; letters (D, V, H and B as well as the gap) in the 3-rd level represent the combinations of any three nucleotides plus the gap; and we use the letter N in the 4-th level to represent all the possible situations. Details of the generalization hierarchy is shown in Figure 2.

Specifically, given two nucleotides $q_{l}^{i}$ and $q_{l}^{j}$ in the *l*-th position of the *i*-th and the *j*-th aligned DNA sequences $Q_{\left(i\right)}$ and $Q_{\left(j\right)}$ , respectively, their obfuscation (nucleotide) code is represented as $g\left(q_{l}^{i},q_{l}^{j}\right)$ . For example, given two aligned nucleotide sequence segments CCTGTAAA and CA-GTRAA, according to the rule in Figure 2, their obfuscation sequence is CMNGTRAA. To measure the information loss after sequence obfuscation, a distance measurement was proposed in (Malin, 2005b). The distance between $q_{l}^{i}$ and $q_{l}^{j}$ after nucleotide obfuscation is defined as:

$$dist\left(q_{l}^{i},q_{l}^{j}\right)=2lev\left(g\left(q_{l}^{i},q_{l}^{j}\right)\right)-lev\left(q_{l}^{i}\right)-lev\left(q_{l}^{j}\right),$$ (2)

where *lev* (⋅) is the level of nucleotides. Based on Eq. 2, the distance between two aligned sequences (suppose the length of both sequences is *L*) can be defined as the sum of distances of all the nucleotides at the same positions, i.e.,

$$d\left(Q_{\left(i\right)},Q_{\left(j\right)}\right)=\overset{L}{\underset{l=1}{\sum }}dist\left(q_{l}^{i},q_{l}^{j}\right).$$ (3)

Using the two sequences CCTGTAAA and CA-GTRAA, according to Eq. 3, we obtain the sequence distance is *d* = 0 + 2 + 4 + 0 + 0 + 1 + 0 + 0 = 7. In our experiments, we use the distance to measure the degree of information loss after sequence obfuscation. Definitely, the shorter the distance is, the less the information loss incurs after sequence obfuscation.

### 2.5 IterMegaBLAST for Genomic Privacy Protection

Given a dataset of DNA sequences, the procedures for our method can be summarized in Algorithm 1. In Algorithm 1, ⌊*x*⌋ means taking the largest integer less than or equal to *x*; ∪ and \ are the set union and set difference, respectively; MegaBLAST $\left(Q_{\left(t\right)},S_{\left(t\right)}\right)$ means using $Q_{\left(t\right)}$ as the query sequence and $S_{\left(t\right)}$ as the searching database to do the MegaBLAST search. Similar to other studies (Li et al., 2012), we set *k* = 2 in our experiments. Note when the number of a dataset is odd, we need to use MegaBLAST to align the last three sequences. After sequence alignment, we obtain the obfuscated sequence for the query sequence and the top homolog. Then we do the second obfuscation on the second top homolog and the obfuscated sequence previously obtained.

For ease of reference, we name our method as IterMegaBLAST, which is publicly available at https://github.com/shibiaowan/IterMegaBLAST.

Algorithm 1 The algorithm for IterMegaBLAST

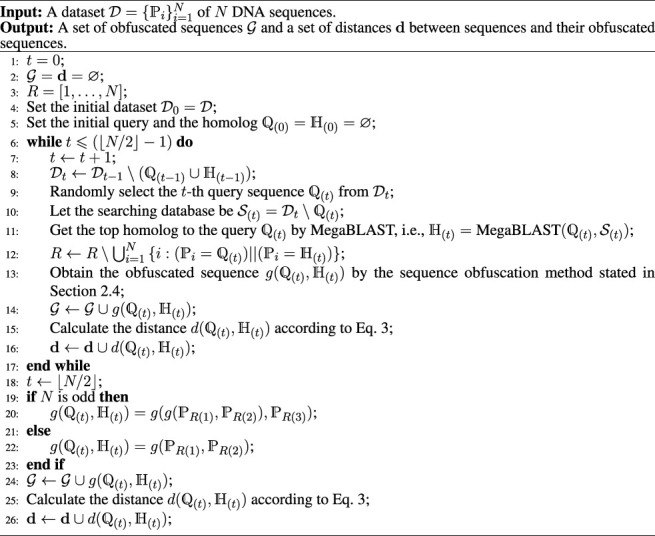

## 3 Results

### 3.1 Datasets

**FIGURE 3:**
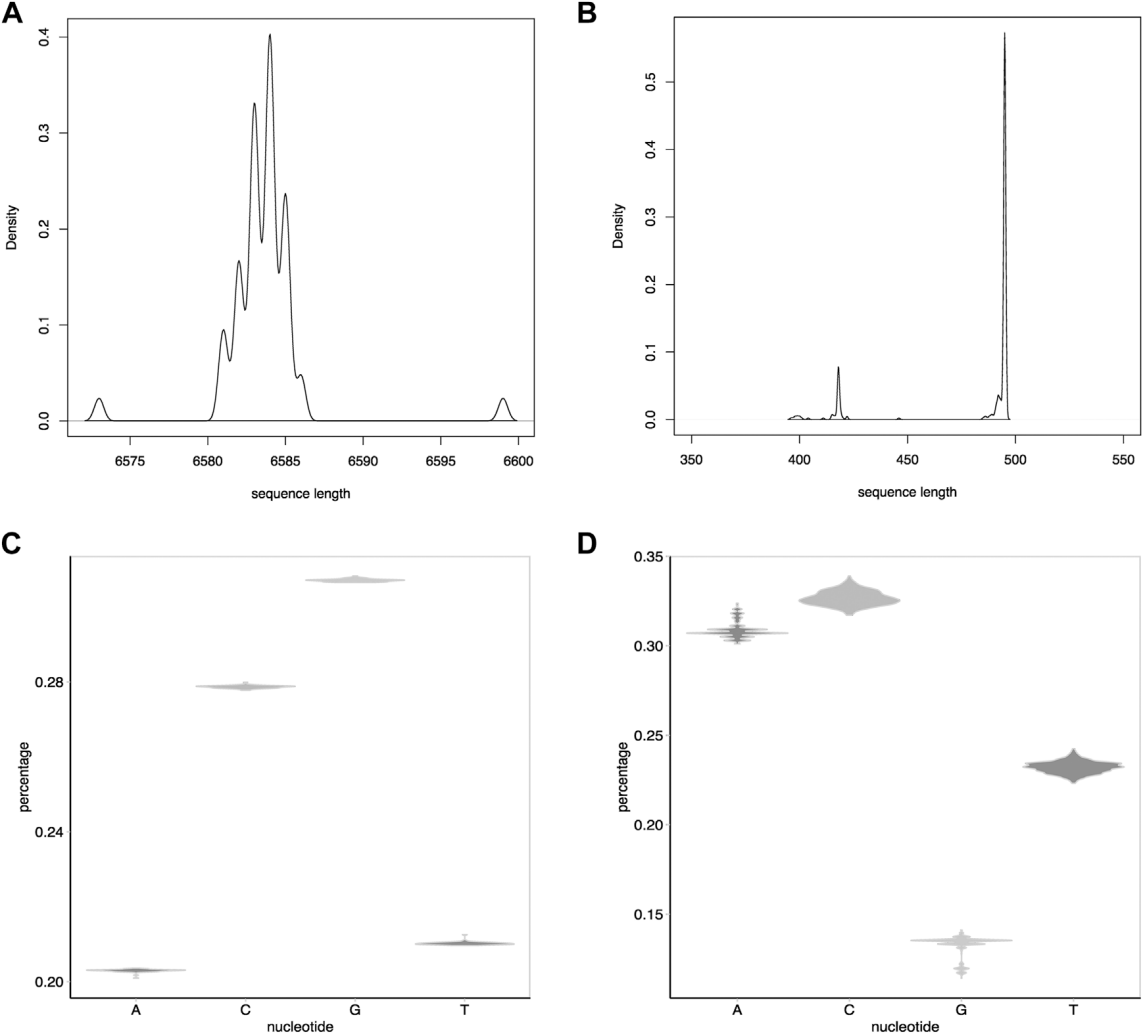
Statistics of the two datasets used in this paper. **(A)** and **(B)**: The density distribution of the sequence lengths for Dataset I **(A)** and Dataset II **(B)**. **(C,D)**: Distributions of the percentages of each nucleotide (i.e., A, C, G, and T) for Dataset I **(C)** and Dataset II **(D)**. The numbers of sequences for Dataset I and Dataset II are 56 and 372, respectively.

Two datasets [Dataset I (Makova et al., 2001) and Dataset II (Yao et al., 2002)] were used to evaluate the performance of IterMegaBLAST. Both datasets are human DNA sequences. Dataset I is a group of DNA sequences in the melanocortin gene promoter region while Dataset II is in the human mitochondrion control region. The numbers of sequences for these two datasets are 56 and 372, respectively. As can be seen from Figure 3A,B, the average sequence length of Dataset I (i.e., 6.58 kb, Figure 3A) is much longer than that of Dataset II (i.e., 0.5 kb, Figure 3B). Besides, the nucleotide G has relatively high enrichment in Dataset I compared to other nucleotides (Figure 3C) whereas Dataset II is enriched in the nucleotide C compared to other nucleotides (Figure 3D).

The average distance between sequences and their obfuscated sequences, and the time complexity were used to measure the performance of different algorithms. Note that because all of the algorithms we compared in this paper are based on the *k*-anonymity, the degree of anonymity (or degree of privacy) (Diaz et al., 2002) should be the same when *k* is the same. Therefore, we do not report the degree of privacy.

### 3.2 Performance of IterMegaBLAST Varying with Respect to the Number of Sequences

**FIGURE 4:**
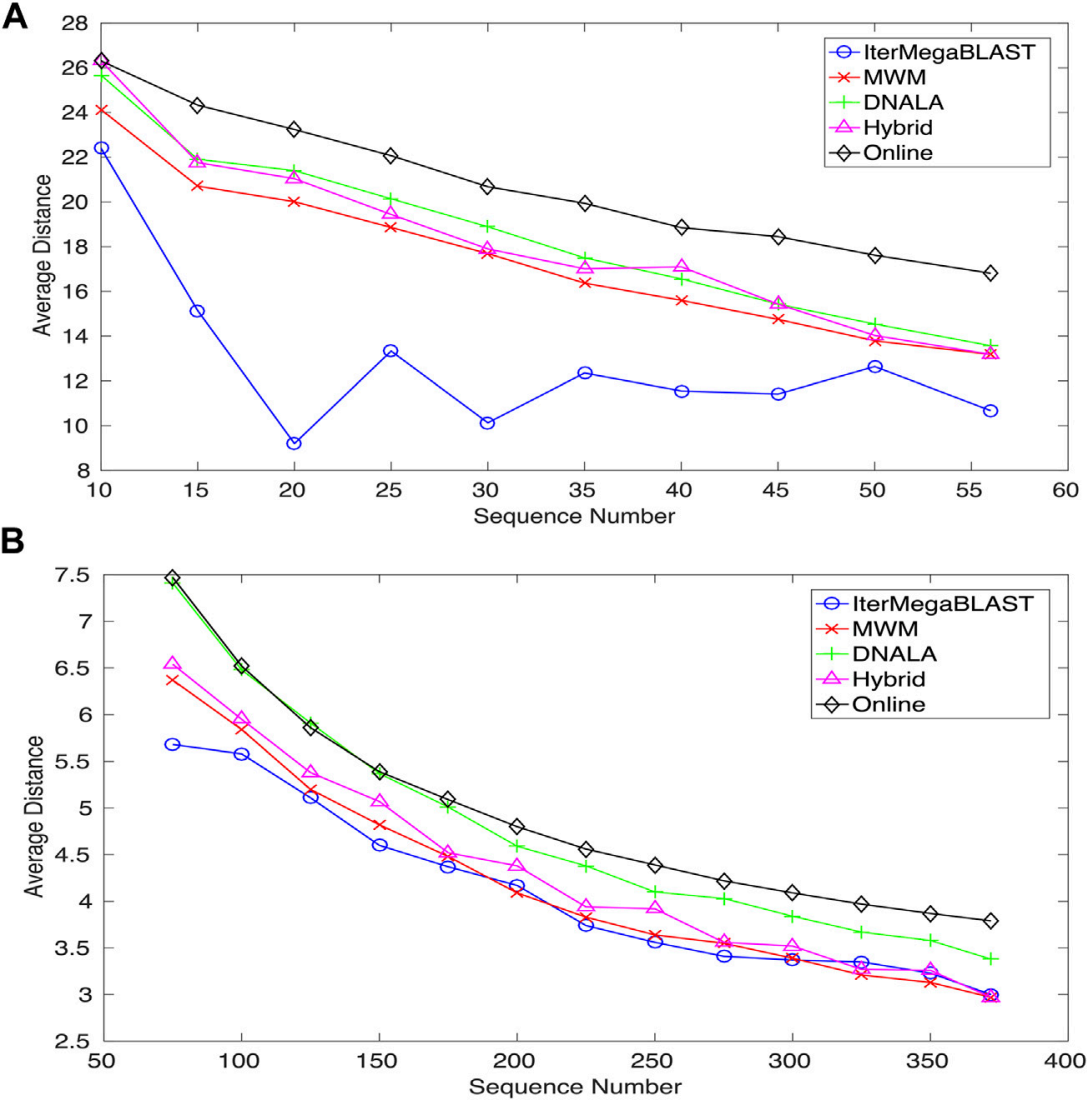
The average distances of IterMegaBLAST varying with respect to the number of DNA sequences for **(A)** Dataset I and **(B)** Dataset II. The shorter the distance is, the less the information loss. DNALA is from (Malin, 2005b), while MWM, Hybrid and Online algorithms are from (Li et al., 2012). IterMegaBLAST is the method proposed in this paper.

Figure 4 compares IterMegaBLAST against several state-of-the-art privacy-protection methods for both Dataset I and Dataset II when the number of DNA sequences gradually increase. DNALA (Malin, 2005b) uses a multiple sequence alignment technique for sequence alignment and uses the CLUSTALW for clustering. All of MWM, Online and Hybrid use global pairwise sequence alignment, while for clustering, they use maximum weight matching (Li et al., 2012), an online algorithm (Li et al., 2012) and hybrid of the former two algorithms. IterMegaBLAST uses an iterative MegaBLAST for both sequence alignment and clustering. The performance is measured by the average distances between sequences and their obfuscated sequences. For readers’ convenience, we have summarized the methodological differences between IterMegaBLAST and other methods in Table 1. Please note that because all of the algorithms we compared in this paper are based on *k*-anonymity for sequence obfuscation, we only show the steps of sequence alignment and clustering in the table. Only DNALA uses a multiple sequence alignment method (MSA) called CLUSTALW whereas other methods use a pairwise sequence alignment method which is generally faster than MSA methods. For the clustering step, MWM has the same time complexity as the greedy algorithm used in DNALA; however, the former is with higher precision. The online algorithm tries to speed up the clustering step based on the MWM method at the expense of less precision. The shorter the distance is, the less the information loss. Because the query DNA sequences for IterMegaBLAST are randomly selected, the performance of IterMegaBLAST may vary a bit even when the same DNA sequences are used. To reduce the bias, we performed IterMegaBLAST ten times for each case (number of sequences). For ease of presentation, only the average performance is shown.

**TABLE 1:** Methodological Comparison between IterMegaBLAST and state-of-the-art genomic privacy-protection methods. PSA: pairwise sequence alignment.

Method	Alignment	Clustering	References
DNALA	CLUSTALW	A greedy algorithm	Malin, (2005b)
MWM	PSA	MWM	Li et al. (2012)
Online	PSA	An online algorithm	Li et al. (2012)
Stochastic hill-climbing	PSA	A stochastic hill-climbing algorithm	Li et al. (2007)
IterMegaBLAST	Iterative MegaBLAST	A MegaBLAST-homolog-based algorithm	This paper

As can be seen from Figure 4A, IterMegaBLAST significantly outperforms all of the state-of-the-art methods in all cases when the number of sequences increases from 10 to 56. While the average distances of all of MWM, DNALA, Hybrid and Online are strictly monotonically decreasing with the number of sequences, this is not the case for IterMegaBLAST, which achieves its best performance when the number of sequences is 20. It is noted that because all of these five methods are based on *k*-anonymity (i.e., *k* = 2), the degree of anonymity (Diaz et al., 2002), which is to measure the degree of how well the privacy is protected, should be the same. Therefore, experimental results suggest that under the same degree of anonymity, IterMegaBLAST can maintain the least information loss for data utility among all the genomic privacy-protection methods. The results also suggest that sequence similarity based methods (i.e., IterMegaBLAST) can provide sufficient privacy protection for genomic data (particularly long DNA sequences) while the information loss maintains at a low level.

Similar conclusions can be drawn from Figure 4B except that IterMegaBLAST may be only comparable to (if not better than) MWM, particularly when the number of sequences is larger than 300. Except MWM, IterMegaBLAST performs better than DNALA and Online for all the ranges of sequence numbers, and outperforms the Hybrid algorithm for all cases except when the number of sequences is around 325. This is probably because the lengths of DNA sequences are vary short (average 0.5 kb) and MegaBLAST is better able to handle long DNA sequences. Moreover, we would like to emphasize that the number of non-standard nucleotides (e.g., N) in the sequences of Dataset II is much larger than that of Dataset I, which contributes to more information loss whereas MegaBLAST treats them with equal weights as those standard nucleotides. On the other hand, MWM directly uses the minimum distance as the criteria to cluster the sequences.

For the genomic-privacy datasets (e.g., Datasets I and II), they are usually with high sequence similarity. When the number of DNA sequences increases, for most of the methods, it is more likely for a query sequence to find its top homolog with higher sequence similarity, thus reducing the distance between the original sequence and their obfuscated sequence. While we observed the similar trend (e.g., Dataset II and the general trend of Dataset I) for IterMegaBLAST, it had a minor difference that it achieved the best performance at 20 instead of further reducing the average distance when the number of sequences further increased for Dataset I. As MegaBLAST is suitable for high-similarity sequence alignment, IterMegaBLAST might form clusters with lower distances between the original sequences and the obfuscated sequences compared to other methods. However, adding more sequences will change the compositions of clusters because more than one homolog might be found with the same high sequence similarity. In this case, by selecting a different homolog to form a cluster with the query sequence, it will affect the alignment of the remaining sequences which might achieve less optimal alignment, leading a bit increase in the average distances. But please note that the general trend of the average distance with respect to the number of sequences is without huge difference between IterMegaBLAST and other methods.

### 3.3 Comparing With State-of-The-Art Methods

To further demonstrate the superiority of IterMegaBLAST, Table 2 compares the performance of IterMegaBLAST against several state-of-the-art privacy-protection methods. Another algorithm called stochastic hill-climbing (Li et al., 2007) is added to compare with IterMegaBLAST. Moreover, DNALA, MWM, Online and Stochastic hill-climbing are capable of performing multiple sequence alignment (MSA) and pairwise sequence alignment (PSA).

**TABLE 2:** Comparing IterMegaBLAST with state-of-the-art genomic privacy-protection methods. *m* ± *n* denotes (mean)±(standard deviation). The performance is measured by the average distance between DNA sequences and their obfuscated sequences. The shorter the distance is, the less the information loss. MSA, multiple sequence alignment; PSA, pairwise sequence alignment.

Dataset	Method	MSA	PSA
I	DNALA Malin, (2005b)	13.79	13.57
	MWM Li et al. (2012)	13.39	13.18
	Online Li et al. (2012)	16.93	16.81
	Stochastic hill-climbing Li et al. (2007)	13.39	13.18
	IterMegaBLAST	**10.78** **±** **0.94**	**10.67** **±** **1.07**
II	DNALA Malin, (2005b)	3.33	3.35
	MWM Li et al. (2012)	2.99	**2.98**
	Online Li et al. (2012)	3.79	3.80
	Stochastic hill-climbing Li et al. (2007)	3.13	3.11
	IterMegaBLAST	**3.05** **±** **0.07**	**3.00** **±** **0.10**

Bold values indicate the best performance.

As can be seen from Table 2, for Dataset I, IterMegaBLAST remarkably outperforms all of the four state-of-the-art methods, no matter they use MSA or PSA; while for Dataset II, IterMegaBLAST performs better than DNALA, Online and stochastic hill-climbing, but its performance is comparable to (if not better than) that of MWM. In other words, under the same degree of anonymity or privacy protection, IterMegaBLAST can achieve higher utilization value compared to other methods.

Table 3 compares the computational time of IterMegaBLAST against MWM equipped with either PSA or MSA. Since MWM performs the best among the four aforementioned methods as demonstrated in the reference (Li et al., 2012), we only report the computational time of MWM here.

**TABLE 3:** Comparing the computational time of IterMegaBLAST with that of state-of-the-art genomic privacy-protection methods. MSA, multiple sequence alignment; PSA, pairwise sequence alignment.

Dataset	Method	Time (seconds)
I	MWM + MSA Li et al. (2012)	>9000
	MWM + PSA Li et al. (2012)	>7000
	IterMegaBLAST	**112**
II	MWM + MSA Li et al. (2012)	>2000
	MWM + PSA Li et al. (2012)	>2000
	IterMegaBLAST	**384**

Bold values indicate the best performance.

As can be seen, IterMegaBLAST performs impressively faster than MWM + PSA and MWM + MSA for both datasets. The reason is that IterMegaBLAST only needs to use MegaBLAST for ⌊*N*/2⌋ times and each time the number of sequences in the searching database will decrease. As we have mentioned, MegaBLAST performs 10 times faster than traditional BLAST, whereas MWM has to obtain all the pair-wise distances for all sequences. Interestingly, the computational time of IterMegaBLAST for Dataset II is much longer than that for Dataset I. This is because the number of sequences in Dataset II is much larger, causing a significantly larger number of MegaBLAST invocations for Dataset II. Moreover, MegaBLAST is more capable of handling long sequences like Dataset I, which also explains why the time advantage of IterMegaBLAST over MWM is more obvious for Dataset I than that for Dataset II.

### 3.4 Example of Using IterMegaBLAST

**FIGURE 5:**
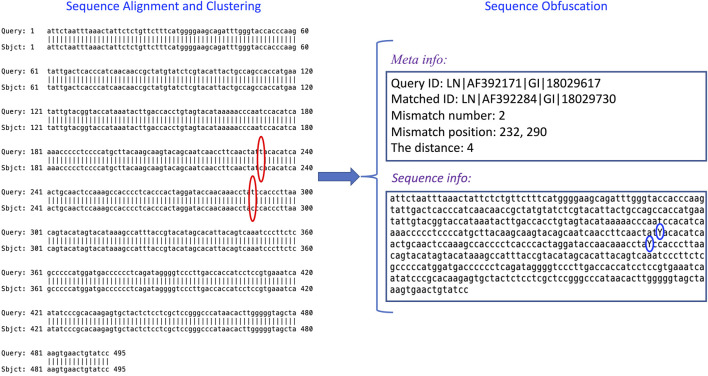
An example of how IterMegaBLAST works. IterMegaBLAST consists of two major steps: sequence alignment and clustering, and sequence obfuscation. Given a query sequence, e.g., LN∣AF392171∣GI∣18029617, IterMegaBLAST first uses MegaBLAST to find its top homolog, e.g., LN∣AF392284∣GI∣18029730, which form a cluster. Then, IterMegaBLAST uses the sequence obfuscation method introduced in Section 2.4 to generate the generalized sequence (see the “Sequence info” box on the right panel) for this cluster. The distance is calculated and the related meta information is produced (see the “Meta info” box on the right panel). The red circles indicate the mismatched nucleotides between the query sequence and the homolog, and the blue circles represent the generalized nucleotides for the mismatches nucleotides.

To further exemplify how IterMegaBLAST is used to protect genomic privacy and minimize the utility loss, we showed an example (Figure 5) of using a query sequence from Dataset II. IterMegaBLAST consists of two major steps: sequence alignment and clustering (the left panel of Figure 5), and sequence obfuscation (the right panel of Figure 5). Specifically, given the query sequence LN∣AF392171∣GI∣18029617, IterMegaBLAST first uses MegaBLAST to find its top homolog, e.g., LN∣AF392284∣GI∣18029730 and a cluster. As can be seen from Figure 5, there are two positions of mismatches, namely 232 and 290 (see the red circles in Figure 5), both of which are “T″ for the query sequence whereas both of the corresponding nucleotides for the homolog are “C”. Then, IterMegaBLAST uses the sequence obfuscation method introduced in Section 2.4 to generate the generalized sequence for this cluster. Thus, the mismatched nucleotides are replaced by the more generalized nucleotide “Y” (see the blue circles in Figure 5). Then, the distance is calculated as 4 according to Eq. 3 and the related meta information is produced. This process can be iteratively performed if more sequences are incorporated and deeper degrees of obfuscation are needed. After obfuscation, it is unlikely to differentiate the query sequence from the sequences in the same cluster, whereas we can keep the other sequence information unchanged to maximize its utility value.

## 4 Discussions

As more and more people are involved in personalized medicine, genomic privacy has become one of the essential yet easy-to-ignore topics. Given multiple national-level projects like “All of Us” and “1 + Million Genomes” Initiative have been proposed across the US and Europe, we expect to see an avalanche of personal genomic data to be sequenced and thus require high-degree of genomic privacy protection. Conventional methods on protecting genomic privacy largely rely on common data privacy methods but ignore the special properties of genomic sequences. As sequence-based data are in in large size and are more complicated than conventional data which are easy to digitalized or vectorized, sequence-specific privacy-protection methods should be proposed to tackle the concern of genomic privacy.

It should also be noted that in recently years, multiple machine learning based methods (Al et al., 2017; Wan et al., 2017; Chen et al., 2020; Carpov et al., 2021) are proposed to balance the tradeoff between data privacy protection and maximize data utilization. Most of these methods will optimize an objective function which maximize the data utility value and simultaneously minimize the privacy compromise. Some of them focus on protecting common data (e.g., demographic data like age, ethnics, address) while other methods focus on protecting genomic-specific privacy. The advantages of machine learning based methods over conventional encryption methods are that it is impossible for a third party to intercept any encryption keys to retrieve the data privacy, although these are achieved at the expense of some degree of data utility loss. Thus, one of the priorities on machine learning based methods are to maximize the data utilization based on the condition that the genomic data privacy has been preserved.

In this paper, we propose a sequence obfuscation method to protect personal genomic privacy by leveraging the properties of DNA sequences and *k*-anonymized method. By sequence alignment and clustering, and sequence obfuscation, we have demonstrated that our proposed method outperform existing state-of-the-art methods in terms of both accuracy and time complexity. It should be also noted that one of the limitations of this paper is that no clear utilization applications have been shown due to the high requirement of specific biological knowledge. Instead, we have demonstrated the effectiveness of our method indirectly from the minimization of the difference between the original sequence data and the encrypted sequence data, i.e., the less the difference, the higher the utilization value of the encrypted data. Although it is logically sound, it might be more impressive to have specific utilization applications like (Gymrek et al., 2013) to demonstrate the applicability of our method. We will dive into this direction in our future research to improve our method on genomic privacy protection.

Besides generalization mentioned in Section 2.3, another common way for data anonymization is data suppression. Suppression is to remove an attribute’s value entirely from a data set. This would be useful when the data features or attributes are clearly defined. For example, the age information for a demographic data, can be suppressed (i.e., removed) from each sample entirely. But please note that the suppression should only be used for features or attributes which are not relevant to the purpose of data utilization. If our purpose is to determine which age groups of people are more inclined to develop a particular disease, removing the age information does not make sense in this case. While for genomic privacy protection, data suppression has not been commonly used because the features in genomic data are not clearly defined. But that does not mean data suppression can’t be applied in genomic data. If we have a specific utilization task in which the genomic features can be clearly defined, the suppression method will be more useful in this case.

In this paper, we used *k* = 2 for the *k*-anonymity in our comparisons. It would be interesting to see how the performance of IterMegaBLAST will be with respect to the increase of *k* in the k-anonymity. However, we would like to emphasize that to have a fair comparison with other methods, we implemented IterMegaBLAST with the same *k* (i.e., *k* = 2) for the *k*-anonymity part. Using different *k*’s will lead to different degrees of privacy protection. Specifically, a larger *k* will yield higher degree of privacy protection at the expense of less data utilization. In other words, the average distances for *k* > 2 will be larger than those for *k* = 2.

As some compared state-of-the-art methods used CLUSTALW for sequence alignment whereas IterMegaBLAST used MegaBLAST, it is interesting to know their differences. First, the major difference between CLUSTALW and BLAST is that CLUSTALW is a multiple sequence alignment tool whereas BLAST is a pairwise sequence alignment (but BLAST can also be adapted to multiple sequence alignment case). IterMegaBLAST is based on MegaBLAST which is similar to BLAST except that MegaBLAST is efficient to handling much longer DNA sequences and it particularly excels in handling highly-similar sequence alignment (which is common for genomic privacy-protection data). Therefore, the major difference between CLUSTALW and IterMegaBLAST is that CLUSTAL is a multiple sequence alignment tool whereas IterMegaBLAST is based on a pairwise sequence alignment tool MegaBLAST. While both of them are popular tools for computing sequence similarity, we believed our algorithm plays a more significant role for improving the performance than the difference between these two tools.

We noted that the two datasets in this paper might be a bit old, thus it might be good to try our method on different datasets to further demonstrate the superior performance. While on another hand, we would like to emphasize that genomic privacy protection is a bit different from traditional machine learning application problems. Traditionally, for machine learning (especially supervised learning) applications, it would be more unbiased when using old data as training sets and using newer data as test sets compared to using old data for both training and test sets. However, in this paper, no supervised learning is involved. Instead, our purpose is to obfuscate the unique properties or characteristics for an individual DNA sequence from a group of highly similar sequences. Using old data will not compromise the unbiasedness of the way we evaluated methods. In our future research, however, we will try our method on larger-scale datasets.

## 5 Conclusion

This paper proposes an accurate and efficient approach, namely IterMegaBLAST, which leverages sequence similarity and information obfuscation for genomic privacy protection. Given a dataset of DNA sequences, we formed clusters by iteratively selecting query sequences and finding their top homologs by MegaBLAST. Subsequently, the aligned sequences in each cluster were obfuscated by replacing the different nucleotides with their lowest common ancestors *via* a DNA generalization lattice scheme. It was found that IterMegaBLAST performs much better than existing genomic privacy-preserving methods with less information loss and higher efficiency under the same degree of genomic privacy protection.

## Data Availability

The data used in this article are publicly available at https://www.ncbi.nlm.nih.gov/pmc/articles/PMC1461732/ and https://pubmed.ncbi.nlm.nih.gov/11953946/.

## Footnotes

1
https://www.genome.gov/about-genomics/fact-sheets/sequencing-human-genome-cost.

2 Re-identification means matching the anonymized personal data with its original information or owner.

3 A homolog is a sequence from a searching database which shares a high sequence similarity with the query one.
